# Acute post-infusion hypokalemia following rituximab therapy in patients with nephrotic syndrome: case series and literature review

**DOI:** 10.3389/fphar.2026.1770911

**Published:** 2026-02-27

**Authors:** Weidong Huang, Lishi Yu, Lie Jin, Fengfen Wu, Yuanyuan Xu, Tingyan Xiang, Wenhui Lei

**Affiliations:** 1 Department of Nephrology, The Fifth Affiliated Hospital of Wenzhou Medical University, Lishui, Zhejiang, China; 2 Department of Rheumatology, The Fifth Affiliated Hospital of Wenzhou Medical University, Lishui, Zhejiang, China; 3 Department of Nephrology, Longquan People’s Hospital, Lishui, Zhejiang, China; 4 Department of Pathology, The Fifth Affiliated Hospital of Wenzhou Medical University, Lishui, Zhejiang, China; 5 Department of Medicine, Liandu District Baiyun Street Community Health Service Center, Lishui, Zhejiang, China

**Keywords:** adverse drug reaction, case report, hypokalemia, nephrotic syndrome, rituximab

## Abstract

**Background:**

Rituximab has become an important therapeutic option for nephrotic syndrome (NS), and its adverse event profile is generally well characterized. However, reports of acute hypokalemia specifically occurring in the post-infusion period remain rare. We aimed to present and analyze cases of this distinct timing of electrolyte disturbance.

**Case Presentation:**

This case series describes two adult patients with NS who developed acute, severe hypokalemia in the hours immediately following a rituximab infusion. Case 1: A 20-year-old male with steroid-dependent minimal change disease developed progressive quadriceps weakness and severe hypokalemia (potassium 1.79 mmol/L) several hours after his fifth rituximab infusion. His previous four infusions had been uneventful. Case 2: A 46-year-old male with membranous nephropathy presented with marked mental fatigue and severe hypokalemia (potassium 2.34 mmol/L) shortly after his sixth cumulative rituximab infusion, following five prior tolerated infusions. Common precipitants of hypokalemia were absent. Both patients responded promptly to potassium supplementation, with full symptomatic recovery and normalization of serum potassium.

**Conclusion:**

Severe hypokalemia can occur acutely after rituximab infusion in NS, even after prior uneventful exposures. Presentations may be delayed and nonspecific. Clinicians should monitor serum potassium before and after rituximab administration to enable timely recognition and management of this rare complication.

## Introduction

Nephrotic syndrome (NS) is a common renal disorder characterized by heavy proteinuria (defined as ≥40 mg/m^2^/hour or a urine protein/creatinine ratio ≥200 mg/mL or urine dipstick protein 3+), hypoalbuminemia (<25 g/L), edema, and hyperlipidemia (Downie et al., 2017). The pathophysiology is complex and involves injury to the glomerular filtration barrier. The incidence of NS varies across regions and populations.

Traditional immunotherapies for NS include glucocorticoids, cyclophosphamide, and calcineurin inhibitors, among others (Boyer et al., 2017; Robinson et al., 2025; Wang et al., 2012). In recent years, biologic agents such as rituximab have been increasingly used in NS, often with favorable efficacy and safety profiles in various settings (Larkins et al., 2024; Rojas-Rivera et al., 2019). Rituximab is a chimeric anti-CD20 monoclonal antibody. CD20 is primarily expressed on pre-B cells and mature B cells; rituximab binds CD20 and activates a cascade of immune effector mechanisms that culminate in B-cell lysis, thereby exerting therapeutic effects (Bergantini et al., 2020). Rituximab is now widely employed in the treatment of autoimmune diseases and malignancies. According to KDIGO 2021 Clinical Practice Guideline for Glomerular Diseases, rituximab is recommended as a first-line therapy for membranous nephropathy (MN) and for minimal change disease (MCD) with frequent relapses or steroid dependence (Teisseyre et al., 2022; Ronco et al., 2021; Rovin et al., 2021). Although there is extensive literature documenting acute and chronic adverse events associated with rituximab, reports of hypokalemia as an adverse event remain scarce.

This study reports two patients with neuroskeletal (NS) involvement who developed hypokalemia following rituximab therapy. The objective is to describe the clinical features of this rare adverse event and to discuss a broad differential diagnosis—not to establish causality—in order to provide safety references that can guide the clinical use of rituximab and enhance pharmacovigilance in this patient population.

## Case presentation

### Case 1

**FIGURE 1:**
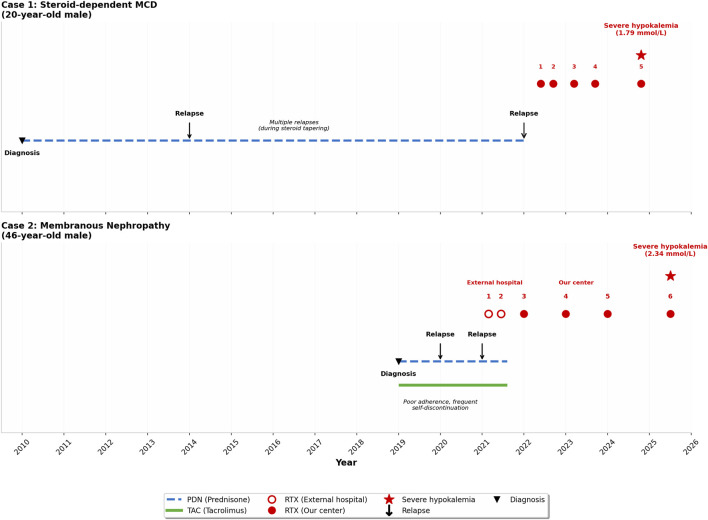
Timeline of immunosuppressive therapy, disease relapses, and rituximab administrations in Case 1 (20-year-old with steroid-dependent minimal-change disease) and Case 2 (46-year-old with membranous nephropathy).

A 20-year-old Asian male presented in 2010 with generalized edema and heavy proteinuria, leading to a first diagnosis of idiopathic nephrotic syndrome (NS) at our center (Figure 1). Because the patient presented in childhood, there was a high risk associated with an percutaneous renal biopsy, and thus a renal biopsy was deferred. He was started on glucocorticoid therapy, which led to resolution of edema and reduction in proteinuria; a low-dose steroid regimen was maintained chronically. In 2014, disease relapse necessitated transfer to a higher-level hospital, where a percutaneous renal biopsy was performed. Pathology was compatible with minimal change disease (MCD), and glucocorticoid therapy was continued.

From initial NS diagnosis in 2010 through 2022, the patient received only steroid therapy, despite repeated recommendations to add immunosuppressants to facilitate steroid tapering and discontinuation. Family economic constraints and the patient’s marked sensitivity to steroids led to repeated refusals of additional immunosuppressive therapy. Consequently, over the ensuing 12 years, disease relapses occurred with steroid dose reductions or withdrawals.

In May 2022, the patient presented with edema and a relapse of proteinuria. Given the cumulative burden and adverse effects of long-term glucocorticoid use (recurrent infections, gastrointestinal intolerance, osteoporosis, and growth retardation), rituximab was initiated as immunosuppressive therapy on 11 May 2022, at a dose of 0.6 g.

**FIGURE 2:**
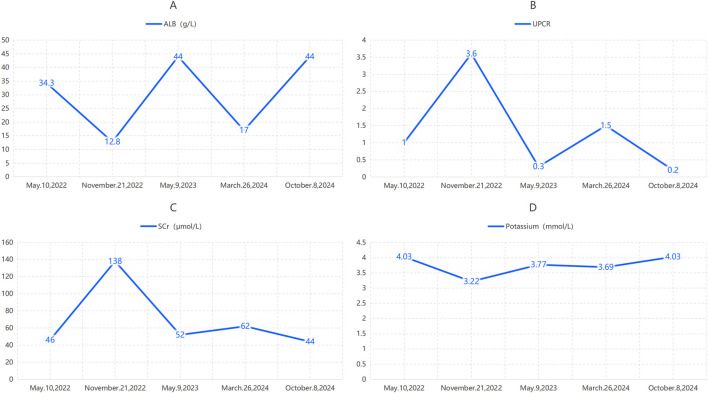
Pre-rituximab laboratory data for Case 1 (injections 1–5). **(A)** Serum albumin; **(B)** Urine protein-to-creatinine ratio (UPCR); **(C)** Serum creatinine; **(D)** Serum potassium. ALB = albumin; Scr = serum creatinine; UPCR = urine protein-to-creatinine ratio.

From May 2022 to October 2024, the patient received five cycles of rituximab infusions. Key laboratory parameters measured prior to each infusion are shown in Figure 2. The first two infusions used 0.6 g per cycle, and the subsequent three used 1 g per cycle. Infusion protocol for the first two cycles: Step 1: Premedication with promethazine HCl 12.5 mg IM and dexamethasone 2.5 mg IV for allergy prophylaxis. Step 2: Rituximab 100 mg in 100 mL normal saline with dexamethasone 2.5 mg, initiated at 15 drops/min. If no allergic reaction occurred within 30 min, the rate was increased by 5 drops/min every 10 min, to a maximum of 40 drops/min, until completion. Step 3: Rituximab 500 mg in 500 mL normal saline, infused at 20–40 drops/min as tolerated, until completion. Infusion protocol for the subsequent three cycles:Step 1: Premedication as above. Step 2: Rituximab 100 mg in 100 mL normal saline with dexamethasone 2.5 mg, initiated at 15 drops/min. If no reaction occurred within 30 min, increase by 5 drops/min every 10 min, to a maximum of 40 drops/min. Step 3: Rituximab 400 mg in 500 mL normal saline, infused at 20–40 drops/min as tolerated. Step 4: Rituximab 500 mg in 500 mL normal saline, infused at 20–40 drops/min as tolerated. All four initial infusions were well tolerated, with no notable allergic reactions, infections, or other adverse events. Post-treatment, nephrotic syndrome remained in partial to complete remission.

On 9 October 2024, the patient received the fifth rituximab infusion (1 g). Pre-infusion laboratory parameters are shown in Table 1. The infusion began at 15:00. Baseline pre-infusion assessment indicated no discomfort, nausea, vomiting, diarrhea, or diuretic use. Vital signs before infusion: temperature 36.4 °C, heart rate 75 bpm, blood pressure 124/68 mmHg.

**TABLE 1:** Laboratory findings on admission.

The laboratory parameters	Case 1	Case 2	Reference range
WBC	6.1	9.1	3.5–9.5 × 10^9^/L
Hb	164	179	130–175 g/L
NEUT%	63.1	70	40.0%–75.0%
AEC	0.08	0.77	0.02–0.52 × 10^9^/L
Plt	377	344	125–350 × 10^9^/L
ALT	10	20	9–50U/L
AST	22	19	15–40U/L
Alb	44.6	25.9	40–55 g/L
BUN	3	4.6	3.1–8.0 mmol/L
Scr	61	63	57–111umol/L
Potassium	4.03	4.14	3.5–5.5 mmol/L
Sodium	137.3	139.9	137–147 mmol/L
Chloride	102.7	112.1	99–110 mmol/L
Calcium	2.53	2.0	2.1–2.5 mmol/L
Magnesium	0.75	0.78	0.75–1.51 mmol/L
Phosphate	1.14	0.95	0.85–1.51 mmol/L
24 h UP	0.2	9.9	<0.15 g/24 h
Absolute Th Count	566	1,063	432–1,341/μL
Absolute Tc Cell Count	1,150	703	238–1,075/μL
Total T Cell Count	1,683	1935	797–2,370/μL
Total B Cell Count	117	5	96–412/μL

Abbreviations: WBC, white blood cells; Hb, Haemoglobin; NEUT%, neutrophil percentage; AEC, absolute eosinophil count; Plt, Platelet; ALT, alanine aminotransferase; AST, Aspartate Aminotransferase; Alb, Albumin; BUN, blood urea nitrogen; Scr, Serum Creatinine; 24 h UP, 24 h Urine Protein; Absolute Th Count, Absolute T-helper Count; Absolute Tc Cell Count, Absolute Cytotoxic T-cell Count; UPCR, Urinary Protein-to-Creatinine Ratio.

The infusion was completed uneventfully at 01:00 on 10 October 2024. Post-infusion vital signs were as follows: temperature 36.0 °C, heart rate 80 bpm, and blood pressure 130/72 mmHg. Subsequently, the patient reported new-onset quadriceps weakness. Although he remained able to ambulate initially, the weakness progressed. Bedside neurological examination showed normal level of consciousness, symmetric strength in all four limbs, and a midline tongue position. Given the progression of weakness, an emergent laboratory recheck revealed severe hypokalemia, with a serum potassium level of 1.79 mmol/L.

**FIGURE 3:**
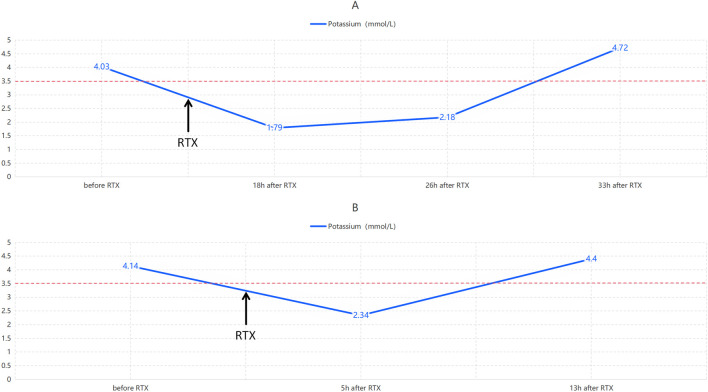
Serial serum potassium with reference range (3.5 mmol/L). **(A)** Potassium changes before and after Case 1, rituximab infusion 5; **(B)** Potassium changes before and after Case 2, rituximab infusion 6. RTX = rituximab. h = hours.

The patient was discharged on 10 October 2024, and was en route home when he developed progressive weakness, rendering him unable to walk independently. He was brought to our emergency department at 14:00 h. Serum potassium at 19:00 h was 1.79 mmol/L, consistent with severe hypokalemia. Immediate management with oral and intravenous potassium supplementation was initiated. Following treatment, serum potassium gradually normalized (Figure 3A), and the patient’s limb weakness markedly improved, with regained ambulation once potassium normalization was achieved.

### Case 2

A 46-year-old Asian male presented 6 years earlier with edema of the lower extremities. At admission, 24-h urine protein excretion was 3.5 g, serum albumin was 23.2 g/L, and anti-phospholipase A2 receptor (anti-PLA2R) antibodies were 525.42 RU/mL. Nephrotic syndrome (NS) was diagnosed, and a percutaneous renal biopsy was performed, revealing stage II membranous nephropathy (MN). Initial therapy comprised glucocorticoids in combination with tacrolimus for immunosuppression, which led to resolution of edema; however, urinary protein fluctuated between 2+ and 3+, and serum albumin rose to a maximum of 30 g/L. The patient demonstrated poor adherence to therapy; after edema resolved, he frequently self-discontinued medications. Relapses occurred, necessitating reinitiation of glucocorticoids and tacrolimus immunosuppression.

Given suboptimal and inconsistent response to traditional immunosuppressive therapy, rituximab was administered at the patient’s two prior encounters (20 March 2021 and 21 May 2021) at 0.6 g per infusion at a higher-level hospital, with no reported adverse effects. Details of the external infusions (infusion rate, total fluid volume, and premedication) were not available.

**FIGURE 4:**
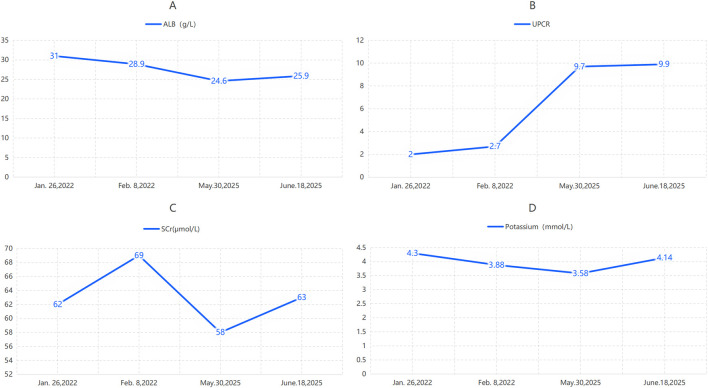
Pre-rituximab laboratory data for Case 2 (injections 3–6). **(A)** Serum albumin; **(B)** urine protein-to-creatinine ratio (UPCR); **(C)** serum creatinine; **(D)** serum potassium. ALB = albumin; Scr = serum creatinine.

From January 2022 to June 2025, the patient received ongoing rituximab immunosuppression at our center. Pre-infusion laboratory parameters are shown in Figure 4. The infusion protocol followed Case 1. The first five infusions were well tolerated, with no reported allergic reactions, infections, or other adverse events.

On 18 June 2025, the patient received the sixth rituximab infusion (1 g). Pre-infusion data are detailed in Table 1. Infusion began at 09:00 in stable condition, with no nausea, vomiting, diarrhea, or diuretic use. Baseline vitals: 36.0 °C, 68 bpm, 130/78 mmHg. The infusion concluded at 17:00 with post-infusion vitals: 36.2 °C, 70 bpm, 134/78 mmHg. The procedure was uneventful.

Shortly after the infusion concluded, the patient reported marked fatigue. In the emergency department, neurological examination showed clear consciousness, fluent speech, and intact cranial nerves. Motor examination revealed proximal muscle strength grade IV and distal strength grade V. Muscle tone was normal, deep tendon reflexes were symmetric, and pathological reflexes were absent. Sensation and coordination were within normal limits. A venous blood gas analysis performed at 22:00 demonstrated severe hypokalemia (serum potassium 2.34 mmol/L). Immediate potassium replacement, both oral and intravenous, was initiated. Serum potassium levels subsequently normalized (Figure 3B), and the patient’s mental status improved concomitantly.

## Discussion

We report two cases of nephrotic syndrome in which severe hypokalemia occurred shortly after rituximab infusion. The first patient had steroid-dependent minimal change disease and developed hypokalemia during his fifth infusion after four prior, well-tolerated courses. The second patient had membranous nephropathy and experienced hypokalemia during his sixth infusion, following five uneventful administrations. The purpose of this report is not to establish causality but to document the temporal association between rituximab infusion and severe hypokalemia and to highlight the broad differential diagnosis required in such scenarios. Rituximab-associated hypokalemia is rare but potentially serious, and its nonspecific presentation may lead to under-recognition. We recommend pre- and post-infusion potassium monitoring to facilitate early detection. Clinicians should remain vigilant for this electrolyte disturbance even in patients who have previously tolerated rituximab without incident.

A review of the existing literature shows that rituximab’s major adverse events include infusion-related reactions (IRRs) (Paul and Cartron, 2019), allergic reactions (Fouda and Bavbek, 2020), infections (McAtee et al., 2021), and posterior reversible encephalopathy syndrome (PRES) (Wardrope et al., 2016). IRRs are the most common events, typically occurring during the first infusion and presenting with fever, chills, fatigue, rash, urticaria, edema, or pruritus (Basu et al., 2018; Levin et al., 2017). Less common manifestations include hypotension, hypertension, myocardial infarction, and hypoalbuminemia (Iijima et al., 2014). While most IRRs are mild to moderate, reports of severe or even life-threatening reactions do exist (Miyabe et al., 2016).

Hypokalemia is a rare but potentially serious adverse effect of rituximab, capable of precipitating arrhythmias or respiratory compromise and is often overlooked. Only a few case reports exist (Table 2). One study described a young woman with steroid-dependent idiopathic nephrotic syndrome who developed acute hypokalemia with dizziness and palpitations after rituximab infusion; intravenous potassium replenishment rapidly resolved symptoms (Guzzi et al., 2020). Another report described two nephrotic syndrome patients who developed symptomatic hypokalemia after rituximab infusion; one patient experienced marked fatigue and myalgias with a substantial drop in potassium, which normalized after aggressive potassium repletion (Song et al., 2023). A case report described successful management with obinutuzumab in a patient who developed acute hypokalemia after rituximab for refractory membranous nephropathy. Switching to obinutuzumab yielded partial renal remission without recurrent hypokalemia, suggesting obinutuzumab as a safe and effective alternative in rituximab-induced electrolyte disturbances (Zhang et al., 2024). Sherwani et al. (2025) reported a case of an autoimmune disease patient without nephrotic syndrome who developed severe hypokalemia (1.3 mmol/L) leading to paralysis after infusion. Miks and Mussarat (2023) described a membranous nephropathy patient who experienced a sudden, severe hypokalemia (2.1 mmol/L) after the second infusion, necessitating ICU admission, highlighting that even patients with prior tolerance may experience life-threatening electrolyte disturbances with subsequent infusions. Collectively, reported cases demonstrate that acute hypokalemia may occur after rituximab infusion in patients with nephrotic syndrome and other autoimmune conditions. Common symptoms include muscle weakness, paralysis, dizziness, palpitations, and fatigue. Hypokalemia was consistently corrected with potassium supplementation. In one patient, switching to obinutuzumab achieved partial renal remission without recurrent hypokalemia, suggesting it as a potential alternative. Recent reports also highlight that severe, life-threatening hypokalemia can occur even after previously tolerated infusions, warranting close clinical monitoring.

**TABLE 2:** Summary of reported cases of hypokalemia following rituximab administration.

Author(s)	Gender	Age (years)	Primary disease	RTX cycle (nth)	RTX dose (mg)	Clinical manifestations	Time to hypokalemia onset	Severity (lowest potassium, mmol/L)	Management	Outcome
Zhang et al. (2024)	Male	31	PLA2R-associated membranous nephropathy	5th	500	Weakness in limbs, palpitations, difficulty moving	30 min after infusion end	1.86	IV potassium chloride + oral potassium citrate	Potassium normalized within 22 h, switched to obinutuzumab
Case 1 (Song et al., 2023)	Male	25	Membranous nephropathy (anti-PLA2R+)	5th	500 (per dose)	Fatigue, inability to stand or move	∼7 h after infusion end	1.8	IV potassium chloride + oral potassium chloride solution	Potassium normalized within 30 h
Case 2 (Song et al., 2023)	Male	75	Minimal change disease	3rd	500	Fatigue, lower limb muscle cramps	2 h after infusion start	2.84	IV potassium chloride + IV calcium gluconate	Symptoms resolved, potassium normalized
Guzzi et al. (2020)	Female	18	Steroid-dependent nephrotic syndrome	6th	500	Dizziness, palpitations, tachycardia	3 h after infusion start	2.3	IV potassium chloride	Potassium normalized within 5 h, ECG abnormalities resolved
Sherwani et al. (2025)	Female	53	Microscopic polyangiitis, Sjögren’s syndrome	4th	1,000	Diffuse muscle weakness, paralysis	3–4 h after infusion	1.3	Oral + IV potassium supplementation	Potassium normalized; symptoms resolved
Miks and Mussarat (2023)	Male	50	Membranous nephropathy	2nd	Not specified	Proximal muscle weakness	5 h after infusion	2.1	Oral + IV potassium repletion, ICU monitoring	Potassium normalized; discharged with oral potassium

RTX: rituximab; PLA2R: phospholipase A2 receptor; IV: intravenous; ECG: electrocardiogram.

RTX, dose refers to the amount administered during the infusion associated with hypokalemia.

Time to hypokalemia onset is reported relative to the start or end of RTX, infusion as documented.

Normal serum potassium range: 3.5–5.0 mmol/L.

All patients recovered normokalemia with potassium supplementation and supportive care.

The pathophysiology of rituximab-associated hypokalemia is not fully understood, but likely involves multiple factors. On one hand, rituximab may disrupt immune regulation after therapy, affecting renal handling of potassium in a way that increases renal potassium loss. The kidney plays a central role in maintaining potassium balance, and transporter and regulatory mechanisms along the nephron are complex. Immune modulation within the renal microenvironment may interfere with epithelial ion transport proteins in the tubular segments, leading to increased potassium excretion and hypokalemia (Abbas et al., 2015). On the other hand, rituximab-induced hypokalemia may be linked to immune-system changes, such as treatment-associated hypogammaglobulinemia, which can elevate infection risk and potentially disturb electrolyte homeostasis (Labrosse et al., 2021; Barmettler et al., 2018). Additionally, rituximab-related inflammatory responses could alter renal ion transport through inflammatory mediators, contributing to potassium distribution and metabolic disturbances that culminate in hypokalemia (Boleto et al., 2018). These mechanisms require further study with larger datasets to be clarified. Studies indicate that rituximab can trigger immune-mediated responses that lead to tubulointerstitial inflammation. This inflammatory process may cause tubular dysfunction and electrolyte disturbances (Boyer-Suavet et al., 2020). Some reports link rituximab-associated hypokalemia to proximal renal tubular dysfunction, suggesting a potential role for tubulointerstitial impairment in this context (Koithara and Makashir, 2025).

Because key laboratory data are missing, the precise mechanism of hypokalemia cannot be determined. Based on available clinical information, several possibilities warrant consideration: intracellular potassium shift (although no typical precipitants were identified, glucocorticoids administered during infusion could contribute); gastrointestinal losses (patients denied vomiting or diarrhea, making this unlikely); medication effects, particularly pre-infusion dexamethasone, whose mineralocorticoid-like activity may confound interpretation; infusion-related dilution (total IV fluid volume was approximately 1,100 mL, but fluid-balance data are unavailable); hormonal causes (hyperaldosteronism cannot be excluded due to missing renin–aldosterone measurements); renal tubular dysfunction (would require urinary electrolytes and acid–base data, which were not obtained); hypomagnesemia (serum magnesium was at the lower limit of normal, making it unlikely as the primary driver); and covert diuretic use (not supported by history). In summary, there is no evidence for common extrarenal potassium losses, severe hypomagnesemia, or undisclosed diuretic use. However, without critical data—acid–base status, fractional excretion of potassium, renin–aldosterone levels, and glucose/insulin measurements—we cannot distinguish among potential mechanisms such as intracellular shift, medication-induced renal potassium wasting, dilution, or tubular dysfunction. Consequently, all mechanistic interpretations remain speculative, and the observed association with rituximab is temporal.

This case series has several important limitations that must be acknowledged. First and foremost, its retrospective design led to the absence of systematically collected, mechanism-defining laboratory data. Critical parameters—such as acid-base status (pH, bicarbonate, anion gap), urinary electrolytes (including potassium and chloride for TTKG calculation), renin and aldosterone levels, serum insulin, and creatine kinase—were not available. This fundamental lack of data prevents any definitive distinction between potential causes of hypokalemia, such as intracellular shift, renal wasting, extrarenal losses, or medication effects, and therefore precludes supporting mechanistic conclusions. Second, the small sample size (two cases) limits the generalizability of the observations. Third, for Case 2, specific details of the earlier rituximab infusions (e.g., exact infusion rates, premedication) were obtained from external records and could not be independently verified. Given these constraints, particularly the missing biochemical data, the association between rituximab infusion and hypokalemia in these cases remains strictly temporal. Any discussion of pathophysiology is necessarily hypothetical and speculative, and no causal inference can be made.

In summary, we report two cases of nephrotic syndrome in which episodes of acute, severe hypokalemia could not be explained by common causes after multiple rituximab infusions. Both cases demonstrated a clear temporal association, yet the absence of key laboratory data leaves the underlying mechanism speculative. These cases underscore the importance of vigilance for electrolyte disturbances during rituximab therapy. Prospective studies should systematically collect relevant biochemical parameters to elucidate potential mechanisms.

## Data Availability

The original contributions presented in the study are included in the article/supplementary material, further inquiries can be directed to the corresponding author.
